# Identifying Hereditary Leiomyomatosis and Renal Cell Cancer through Unobtrusive Cutaneous Nodules: A Clinical Report

**DOI:** 10.15586/jkc.v12i1.374

**Published:** 2025-01-27

**Authors:** Emilija Šeštokaitė, Eglė Preikšaitienė, Justas Arasimavičius

**Affiliations:** 1 Clinic of Infectious Diseases and Dermatovenerology, Vilnius University Faculty of Medicine, Vilnius, Lithuania;; 2 Department of Human and Medical Genetics, Institute of Biomedical Sciences, Faculty of Medicine, Vilnius University, Vilnius, Lithuania

**Keywords:** fumarate hydratase, HLRCC, hereditary renal cell cancer, leiomyomatosis, renal cell carcinoma

## Abstract

Cutaneous leiomyomas (CLMs) are associated with Hereditary Leiomyomatosis and Renal Cell Cancer (HLRCC) syndrome (Mendelian Inheritance in Man [MIM]: 150800)—a rare genodermatosis caused by a heterozygous pathogenic variant in the fumarate hydratase (*FH*) gene. It is characterized by a predisposition to develop cutaneous and/or uterine leiomyomas and an aggressive type of renal cell carcinoma (RCC). We describe a 27-year-old male who presented with a painful nodule on the left upper arm persisting for 5 years and the subsequent emergence of painless nodules in various parts of the body over the past two years. A family history of RCC prompted suspicion of the HLRCC syndrome. Cutaneous examination revealed erythematous subcutaneous nodules, with histological analysis confirming CLM. Genetic testing identified a pathogenic variant in the *FH* gene, confirming the diagnosis of HLRCC. Management involved surgical excision of the symptomatic nodules and genetic counselling/testing for the proband and his family members. The long-term follow-up plan includes dermatological and nephrological surveillance with annual renal magnetic resonance imaging (MRI) scans. This report aims to enhance the awareness of this disease and highlight the role of cutaneous lesions in facilitating early detection.

## Introduction

Cutaneous leiomyomas (CLMs) are rare tumors originating from the smooth muscle tissue and are linked to disorders like the hereditary leiomyomatosis and renal cell cancer (HLRCC) syndrome (1). HLRCC is caused by inactivating pathogenic variants in the fumarate hydratase (*FH*) gene, inherited in an autosomal dominant manner (2, 3). This syndrome predisposes individuals to develop leiomyomas of the skin and/or uterus, along with biologically aggressive renal cell carcinoma (RCC) with papillary features (4, 5). Thus, despite its rarity, HLRCC poses a significant risk of mortality. As CLMs are typically the initial presentation of this disease, dermatologists can be instrumental in early detection and referral, thereby offering patients the opportunity for best treatment outcomes. This report aims to enhance the awareness of this disease and highlight the role of cutaneous lesions in facilitating early detection.

## Clinical Report

**Figure 1:**
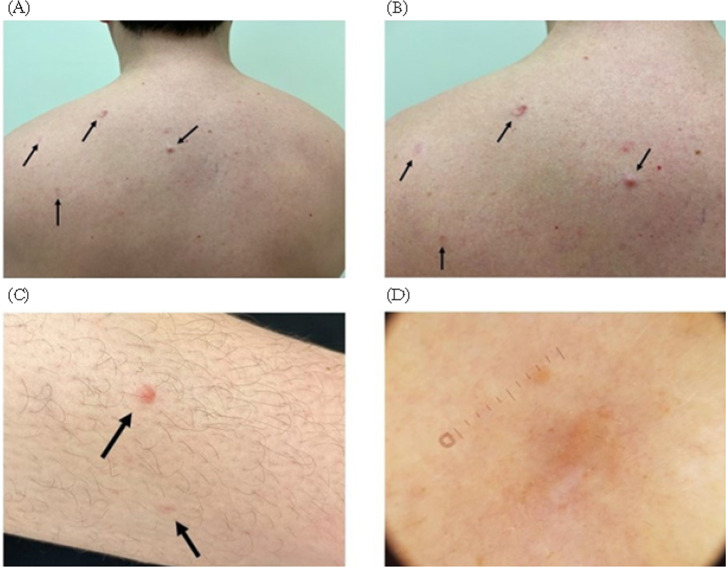
(A) Multiple 3–6 mm diameter erythematous subcutaneous nodules (black arrows) located on the shoulder girdle of the patient. (B) An enhanced view of nodules on the shoulder girdle. (C) Analogous lesions on the right calf. (D) Dermoscopy showed a central hypopigmented area with a slight peripheral hyperpigmentation on one side and some erythematous areas with fine ectatic vessels.

**Figure 2:**
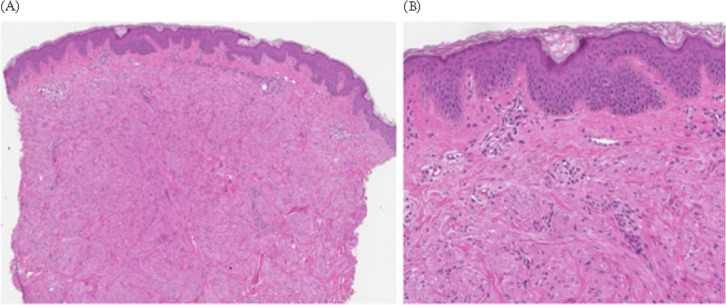
Pathology of subcutaneous nodule. (A) A tumor in the dermis (H&E stain, x40) formed by (B) elongated cells with eosinophilic cytoplasm, central, long blunt-edged, monomorphic, mitotically inactive nuclei (H&E stain, x100).

A 27-year-old male presented to the dermatology clinic complaining of a painful nodule in his left upper arm, which had been bothering him for 5 years. Patient reported that over the past two years, new nodules had begun to appear in other areas of his body, including the shoulder girdle, right arm, and calf, though these were painless. The patient indicated that the lesions first manifested at the age of 18. Upon further questioning, he recounted that his sister had been diagnosed with RCC at 27 years of age, which had a lethal outcome at 28. Furthermore, the patient also recalled the presence of similar cutaneous nodules on his father. Skin examination revealed the presence of erythematous subcutaneous nodules ranging in size from 3 mm to 6 mm at aforementioned sites (Figure 1). A 3 mm punch biopsy was taken from the erythematous nodule in the shoulder girdle and sent for histological examination. The pathological analysis of the biopsy specimen revealed typical histological features consistent with CLM (Figure 2). Due to suspicion of a cancer predisposition syndrome, the patient was referred for genetic counselling. Exome sequencing was conducted on the proband’s genomic deoxyribonucleic acid (DNA) using the HumanCore Exome Kit (Twist Bioscience), as previously described (6). Genes associated with solid tumors were analyzed and the pathogenic variant in *FH* gene NM_000143.3:c.698G>A, NP_000134.2:p.(Arg233His), rs121913123 was identified, thus confirming the diagnosis of HLRCC. A subsequent renal ultrasound did not yield any suspicious findings, and the patient’s treatment plan comprised surgical excision of the painful leiomyomatous nodules, followed by genetic counselling and testing for the patient’ parents and nephew. In the case of our patient, the long-term management plan involves annual follow-ups with a dermatologist and nephrologist, alongside yearly renal magnetic resonance imaging (MRI) scans. In the event of suspicion of a renal neoplasm, prompt surgical intervention will be undertaken.

## Discussion

Blum and Jean linked leiomyomas of the skin with uterine leiomyomas (ULMs) for the first time in 1954, and later in literature, it was referred to as “Reed’s syndrome” as first reports of this condition emerged (5). In 2001, the association between leiomyomatosis and renal cell cancer was discovered in two Finnish families, and therefore the term Hereditary Leiomyomatosis and Renal Cell Cancer was established (1, 7). Since then, over 200 affected families have been reported, yet the prevalence of the syndrome is unknown and is believed to be underdiagnosed due to significant intra- and interfamilial phenotype variation (1, 5).

CLMs are the earliest and most prevalent manifestation of HLRCC, ultimately leading to a diagnosis. In 71–100% of individuals with heterozygous *FH* pathogenic variant, CLMs occur in early adulthood and tend to increase in number and size with age (4, 5). They present as benign, firm, smooth papulonodules, ranging in color from skin-colored to light brown, frequently affecting the face, neck, trunk, and extremities. Larger lesions in particular are known to cause pain or itching (1). Surgical excision is the standard course of treatment for symptomatic lesions (5).

ULMs affect between 81.7% and 98% of females with the *FH* heterozygous pathogenic variant (1, 4). Although the most common benign pelvic tumor in females, those diagnosed with HLRCC typically have more uterine fibroids and develop them on average 10 years earlier than the general population (mean 30) (4, 5). ULMs pose a significant risk to fertility, and due to their heavily symptomatic nature, they often necessitate early hysterectomies (4, 5). Chayed *et al*. found that 77.2% of women with HLRCC undergo removal of the uterus at a mean age of 34.7 (4); however, the potential for malignant transformation of these fibroids remains a topic of debate (3).

While CLMs and ULMs are often symptomatic, the true morbidity of the syndrome is linked to RCC (4). Despite a penetrance of approximately 15%, RCC is typically a biologically aggressive early metastatic tumor that can manifest at a young age (mean 41 years, earliest report at 11 years) (8). These tumors are typically unilateral and solitary, and type 2 papillary histomorphology is the most commonly encountered type (5). The life expectancy of HLRCC syndrome depends on meticulous surveillance for early signs of RCC. The median survival period for individuals with metastatic RCC is 18 months (5). Magnetic resonance imaging is the preferred method for screening (9), as the type 2 papillary renal carcinoma is often isoechoic on ultrasound and can metastasize despite a small primary tumor size (4, 8).

This report seeks to show the role of a detailed skin evaluation for early diagnosis of rare systemic disorders. The patient presented with only a cutaneous manifestation prior to the diagnosis of HLRCC, which is unique, as the majority of the reported HLRCC presentations involve patients with two or more manifestations that prompt suspicion of this syndrome. This discrepancy may be attributed to the predominance of female patients in HLRCC cases, as both CLMs and ULMs are known to have high penetrance. Additionally, women may also be more inclined to seek medical attention for skin lesions. This could indicate a potential underdiagnosis of men, as they typically present only two manifestations: CLMs and renal cell cancer. CLMs are frequently too inconspicuous to prompt suspicion in patients, while renal malignancy, though more noticeable, is often diagnosed too late to yield a favorable outcome.

A single comparable HLRCC report by McKelvey *et al*. of a 31-year-old male presenting with solely cutaneous symptoms was identified. However, this patient was referred due to fertility issues. The authors argue that male infertility should be recognized as a manifestation of HLRCC, given that fumarase has previously been implicated in sperm number and motility (10). Therefore, this issue may warrant further investigation in our patient.

It is recommended that all patients and at-risk family members undergo regular clinical surveillance with an emphasis on early detection of RCC. It should comprise an annual skin examination from the time of diagnosis to monitor for any changes. In addition, an annual gynecological consultation should be undertaken from the age of 20 to assess the severity of uterine fibroids. Last, an annual contrast-enhanced renal MRI should be performed, starting from the age of 8 (4, 5). Early detection and surgical excision at the first sign of HLRCC-associated kidney tumors rather than active surveillance are critical.

## Conclusion

HLRCC is a rare syndrome, but diagnosing it is of vital importance for patients and their families. In our patient, the first symptom that prompted suspicion for HLRCC was cutaneous nodules, later supported by a family history of RCC. This emphasizes the role of a dermatologist for early detection and the need to explore the patient’s familial history of CLMs, ULMs (including hysterectomies), and RCCs. Genetic testing plays a crucial role in the management of these patients and serves as the referral point to the multitude of disciplines involved in their care.
